# The Early Diagnosis of Perforated Gastric Ulcer

**Published:** 1907-08

**Authors:** 


### THE EARLY DIAGNOSIS OF PERFORATED GASTRIC ULCER.

Harnett observes that in difficult and doubtful cases, before making a diagnosis, the following acute abdominal condition must be considered and excluded: Perforated duodenal ulcer; ruptured ectopic gestation sac; acute perforating appendicitis; acute intestinal obstruction; acute pancreatitis. The diagnosis of perforated duodenal ulcer is very difficult, since this condition, up to recently, has been itself rather obscure, and a diagnosis of perforation rarely made ante mortem or previous to operation. Ruptured ectopic gestation sac. This condition occasionally simulates perforation of a gastric ulcer. Acute perforating appendicitis. A perforation of the appendix may occur suddenly, the first symptoms being coincident with the onset of the perforation. In these cases the patient is suddenly seized with pain in the right iliac fossa, accompanied by collapse, vomiting, rigidity, first confined to right side of abdomen, but soon becoming general; distention with paralysis of lower bowel and tympanites. In these cases, when seen early, local tenderness and resistance and muscular rigidity are the most valuable diagnostic signs, the pain in the right iliac fossa being deceptive and often simulated by other conditions. Acute intestinal obstruction. In this condition, usually due to an internal strangulation, the patient is generally in perfect health when suddenly seized with acute abdominal pain, usually referred to the umbilicus or epigastrium. There is marked collapse and pallor, and vomiting soon sets in, which later becomes bilious and finally stercoraceous. The abdomen is at first flaccid, and there is no marked tenderness on pressure, distention coming on gradually. The pain is usually paroxysmal, and vomiting may be considered as a most valuable diagnostic sign. Acute pancreatitis. This condition is rarely diagnosticated during life, although successful treatment has been recorded in a few instances. The symptoms closely simulate those of acute peritonitis—viz. intense pain and vomiting, accompanied by severe collapse, frequent pulse, marked tympanites, with some pyrexia, and a variable amount of constipation.—Dublin Journal Med. Science, June, 1907.

